# Exploration of Local Wisdom of the Archipelago Region to Design Ethno-STEM Project-Based Learning Modules for Ecological Awareness Learning

**DOI:** 10.12688/f1000research.178677.1

**Published:** 2026-03-18

**Authors:** Sundari Hamid, Ahmad Swandi, Amri Amal, Muhammad Ilham S, Arwin Arif

**Affiliations:** 1 Primary Teacher Education, Universitas Bosowa, Makassar, South Sulawesi, 90231, Indonesia; 2 Educational Science, Postgraduate Program, Universitas Negeri Makassar, Makassar, South Sulawesi, 90222, Indonesia; 3 Educational Science, Postgraduate Program, Universitas Negeri Makassar, Makassar, South Sulawesi, 90222, Indonesia; 4 Educational Science, Postgraduate Program, Universitas Negeri Makassar, Makassar, South Sulawesi, 90222, Indonesia; 5 Educational Science, Postgraduate Program, Universitas Negeri Makassar, Makassar, South Sulawesi, 90222, Indonesia

**Keywords:** Ethno-STEM education, Project-Based Learning, Ecological awareness, Local wisdom integration, Archipelagic education

## Abstract

**Background:**

Ecological awareness among students in archipelagic regions is shaped by local environmental conditions and cultural practices. Integrating local wisdom into science instruction may provide a contextually relevant foundation for strengthening students’ ecological understanding and behaviors. This study aimed to explore students’ ecological awareness and to use the findings as a basis for designing an Ethno-Science, Technology, Engineering, and Mathematics Project-Based Learning (Ethno-STEM PjBL) module.

**Methods:**

An exploratory and design-oriented approach was employed. Ecological awareness was profiled across four domains: sustainable consumption, energy use, waste management, and water conservation. Data was collected from 63 junior secondary students in an archipelagic region using validated questionnaires and interviews. Descriptive analysis was conducted to identify domain-specific patterns and contextual learning needs. An Ethno-STEM PjBL module was developed through a diagnostic-based design framework integrating ecological awareness data, local wisdom, and Science, Technology, Engineering, and Mathematics principles. Expert validation was conducted using content validity indices to assess feasibility.

**Results:**

The findings indicate uneven ecological awareness across the four domains, suggesting that environmental attitudes and behaviors are influenced by contextual and cultural factors. These results informed the development of a context-responsive Project-Based Learning module that systematically aligns local wisdom with domain-specific ecological needs. Expert validation indicated high feasibility (content validity indices >0.90 across components).

**Conclusions:**

This study presents a data-informed and context-sensitive framework for designing Ethno-Science, Technology, Engineering, and Mathematics Project-Based Learning modules grounded in ecological awareness profiling. The approach demonstrates how local wisdom can be systematically integrated into instructional design to support culturally responsive science learning in archipelagic contexts.

## 1. Introduction

Education plays a strategic role in preparing younger generations to respond to increasingly complex environmental challenges. Global environmental problems such as climate change, marine pollution, waste accumulation, and freshwater scarcity are no longer abstract issues but realities that directly affect human life, particularly in vulnerable regions such as small islands and archipelagic areas ( UNESCO, 2016, 2017). In many island contexts, water insecurity, limited waste management infrastructure, and climate-related risks have been identified as key constraints to sustainable living ( Greening the Islands, 2024; SMILO, 2023; UNESCO Jakarta, 2023). Consequently, students living in archipelagic regions grow up in environments where ecological issues are not distant phenomena but part of their daily experiences. This condition highlights the importance of education that not only delivers scientific knowledge but also fosters a deep sense of ecological awareness rooted in local realities within the framework of Education for Sustainable Development (ESD) ( UNESCO, 2016, 2025).

In archipelago regions, environmental challenges are shaped by unique geographical, social, and cultural contexts. Issues such as marine waste accumulation, dependence on imported food and energy, limited access to clean water, and vulnerability to climate-related risks are commonly encountered ( Greening the Islands, 2024; SMILO, 2023). At the same time, island communities often possess rich local wisdom related to sustainable resource use, food processing, waste handling, and water management that has been passed down through generations ( Alkhudri et al., 2024; Hamna & Ummah, 2024). However, these forms of local knowledge are rarely integrated systematically into formal school learning. As a result, students may learn environmental concepts in an abstract manner, disconnected from the ecological practices and challenges that surround them. This gap between school learning and lived environmental realities can limit the development of meaningful ecological awareness among students.

Ecological awareness is increasingly recognized as a core competency for education in the twenty-first century, particularly within the ESD agenda. Ecological awareness refers to an individual’s understanding of the relationship between human activities and environmental systems, accompanied by concern, responsibility, and willingness to engage in environmentally responsible behavior ( UNESCO, 2016; Cruz & Manata, 2020). Rather than being a single-dimensional construct, ecological awareness is commonly conceptualized as multidimensional, encompassing cognitive, affective, and behavioral components that together support responsible environmental action ( UNESCO, 2016; Hines et al., 1987). Students are expected not only to understand environmental concepts but also to develop positive attitudes toward environmental protection and to translate this understanding into everyday actions ( Bock & Wickings, 2025; Babintseva et al., 2025).

In this study, ecological awareness is conceptualized through four domain-specific dimensions that are closely connected to students’ daily lives in archipelagic contexts: consumption and carbon footprint, renewable energy, waste management, and water conservation (Dipalaya, 2024). These domains reflect critical environmental issues faced by island communities, such as dependence on imported goods, energy insecurity, waste disposal challenges, and limited freshwater availability ( Greening the Islands, 2024; SMILO, 2023). Focusing on these domains allows ecological awareness to be examined in a contextual and concrete manner, rather than as a generalized attitude detached from real-life practices. Understanding students’ ecological awareness across these domains is essential for designing learning experiences that are both relevant and transformative.

To foster ecological awareness effectively, learning approaches must move beyond conventional teacher-centered instruction and engage students in authentic, meaningful activities. Project-Based Learning (PjBL) has been widely acknowledged as an instructional approach that supports active learning, problem-solving, and higher-order thinking by involving students in extended investigations of real-world problems ( Guo et al., 2020; López et al., 2024). When combined with Science, Technology, Engineering, and Mathematics (STEM) education, PjBL offers opportunities for students to apply interdisciplinary knowledge to design solutions for environmental challenges ( Pertiwi et al., 2024). Empirical studies have shown that STEM-PjBL can enhance environmental literacy, pro-environmental attitudes, and environmentally responsible behaviors more effectively than conventional instruction ( Azrai et al., 2024; Karnoto, 2025; Pertiwi et al., 2024).

Ethno-STEM Project-Based Learning emerges as a pedagogical approach that addresses the limitation of generic STEM projects by integrating STEM concepts with local wisdom and cultural practices ( Sumarni & Kadarwati, 2020; Martawijaya et al., 2023a; Sudarmin et al., 2023). Through Ethno-STEM, scientific learning is contextualized within the cultural and ecological knowledge of local communities, allowing students to perceive science as relevant to their own lives ( Nugraheni, 2023; Martawijaya et al., 2023b). In archipelagic settings, Ethno-STEM PjBL enables students to explore environmental issues such as food consumption patterns, renewable energy use, waste management, and water conservation through projects grounded in local practices and materials ( Nugraheni, 2023; Hamna & Ummah, 2024). This integration not only enhances conceptual understanding but also strengthens students’ sense of ownership and responsibility toward their environment.

Despite the growing body of research on Ethno-STEM and project-based learning, most existing studies focus on measuring learning outcomes or evaluating the effectiveness of instructional interventions ( Martawijaya et al., 2023b; Yusoff, 2019). Relatively few studies begin by systematically examining students’ ecological awareness profiles as a basis for instructional design, particularly in island or archipelagic contexts ( Alkhudri et al., 2024; Kim & Cho, 2022). As a result, many learning modules are developed without sufficient consideration of which aspects of ecological awareness require the most attention or support. This gap suggests the need for an exploratory approach that first maps students’ ecological awareness across relevant domains before designing learning materials intended to strengthen it.

Developing learning modules based on students’ ecological awareness profiles offers a more responsive and evidence-based approach to environmental education. By identifying which domains of ecological awareness are relatively strong and which remain underdeveloped, educators can design learning activities that address students’ actual needs rather than assumed deficiencies ( Cruz & Manata, 2020; Yusoff, 2019). In archipelagic regions, such an approach is particularly important, as environmental behaviors are often shaped by long-standing habits, cultural norms, and structural constraints ( Hines et al., 1987; Bamberg & Möser, 2007). Learning modules that are sensitive to these conditions have greater potential to foster meaningful and sustainable changes in students’ ecological awareness and behavior.

This study does not aim to propose a universal instructional model, but rather a context-responsive Ethno-STEM PjBL design framework grounded in ecological awareness profiling. Accordingly, this study aims to (1) explore students’ ecological awareness profiles across four domain-specific dimensions in an archipelagic context, and (2) design an Ethno-STEM Project-Based Learning module based on these profiles as a data-driven and context-sensitive instructional framework. This study contributes to the field by (1) developing a domain-based ecological awareness profiling framework for students in archipelagic contexts, (2) introducing a data-driven instructional design model for Ethno-STEM Project-Based Learning grounded in empirical ecological awareness data, and (3) integrating local wisdom into a structured diagnostic-based module development approach that bridges cultural knowledge and STEM education.

## 2. Method

### 2.1 Research design

This study employed an exploratory research design followed by a development approach ( Zamanzadeh et al., 2015; Yusoff, 2019). The exploratory phase was conducted to obtain an in-depth understanding of students’ ecological awareness profiles in an archipelagic context, while the development phase focused on designing an Ethno-STEM Project-Based Learning (PjBL) module based on the results of this exploration. This design was chosen to ensure that the developed learning module was grounded in empirical data regarding students’ actual ecological awareness rather than based on theoretical assumptions alone.

The exploratory approach allowed the researchers to map domain-specific ecological awareness across key environmental issues relevant to island communities. The findings from this phase were then used as the primary reference for determining learning priorities and structuring project activities within the Ethno-STEM PjBL module. Following the module design, an expert validation process was conducted to evaluate the feasibility, clarity, and contextual relevance of the developed module before further implementation or effectiveness testing.

### 2.2 Participants and research context

The study was conducted in a junior high school located in an archipelagic region of South Sulawesi, Indonesia. The research site represents a typical small-island educational context characterized by close interaction with coastal and marine environments, limited educational infrastructure, and strong local cultural traditions related to resource use and environmental management. These characteristics make the site particularly relevant for exploring ecological awareness and developing context-sensitive learning materials.

Participants in the exploratory phase consisted of 63 junior high school students enrolled at the selected school. All participants were students who regularly interacted with environmental conditions typical of island communities, such as marine waste, limited freshwater resources, and dependence on external energy and food supplies. The selection of participants was based on their availability and willingness to take part in the study, as well as their relevance to the research objectives. Table 1 shows the demographics of the participants in this study.

**Table 1.** Participant demography.

Demography		N	Percentage (%)
**Gender**	Male	34	54
	Female	29	46
**Class**	7	14	22
	8	25	40
	9	24	38

In the development phase, expert validators were involved to assess the quality of the Ethno-STEM PjBL module. The validators consisted of experts in science education, environmental education, and instructional design. Their role was to evaluate the module from pedagogical, content, and contextual perspectives to ensure its suitability for use in archipelagic school settings.

### 2.3 Research instrument

The primary instrument used in the exploratory phase was an ecological awareness questionnaire designed to measure students’ awareness across four domain-specific dimensions relevant to archipelagic environments: (1) consumption and carbon footprint, (2) renewable energy, (3) waste management, and (4) water conservation ( Dipalaya et al., 2024). These domains were selected to reflect key environmental challenges commonly experienced by island communities and to capture ecological awareness in a concrete and contextual manner.

Each domain encompassed cognitive, affective, and behavioral aspects of ecological awareness ( UNESCO, 2016; Hines et al., 1987). Cognitive aspects measured students’ understanding of environmental concepts, affective aspects captured attitudes and concern toward environmental issues, and behavioral aspects assessed self-reported tendencies to engage in environmentally responsible actions. The questionnaire items were rated using a Likert-type scale, allowing students to express degrees of agreement with statements related to daily environmental practices and beliefs which is a common approach for measuring latent constructs such as environmental concern and ecological awareness ( Cruz & Manata, 2020; Masriah et al., 2025).

In the development phase, an expert validation sheet was used to evaluate the Ethno-STEM PjBL module. The validation instrument covered several aspects, including content relevance, alignment with ecological awareness domains, clarity of project instructions, integration of local wisdom, and feasibility of implementation in island school contexts. The validation instrument covered several aspects. Content validity indices (I-CVI and S-CVI) were calculated to quantify experts’ agreement on the relevance and clarity of each item ( Zamanzadeh et al., 2015; Yusoff, 2019).

### 2.4 Data collection procedure

Data collection was conducted in two main stages corresponding to the research design. During the exploratory stage, students were asked to complete the ecological awareness questionnaire under the supervision of the researchers and classroom teachers. Prior to questionnaire administration, students were informed about the purpose of the study and assured that their responses would be used solely for research purposes.

The results of the questionnaire were then analyzed to identify patterns and variations in ecological awareness across the four domains. These findings informed the development stage, in which the Ethno-STEM PjBL module was designed. The module development process involved identifying relevant local environmental issues, mapping each project activity to specific ecological awareness domains, and integrating STEM concepts with local cultural practices and materials.

Following module development, the expert validation stage was conducted. The draft module was distributed to expert validators, who reviewed and assessed it using the validation sheet. Feedback from the validators was collected and used to refine the module to improve clarity, contextual relevance, and pedagogical coherence.

### 2.5 Data analysis

Data from the ecological awareness questionnaire were analyzed using descriptive statistical techniques. Descriptive statistics are widely used in environmental awareness research to profile students’ baseline levels across domains before designing or implementing interventions ( Cruz & Manata, 2020). Mean scores were calculated for each ecological awareness domain to determine students’ overall awareness levels. These scores were then categorized to identify domains with relatively high, moderate, or low levels of ecological awareness. The results of this analysis served as the basis for determining priority areas in module design.

Data from the expert validation process were analyzed using quantitative and qualitative approaches. Quantitatively, validity indices were calculated to determine the level of agreement among experts regarding the feasibility of the module. Qualitatively, experts’ comments and suggestions were examined to identify aspects of the module requiring revision or improvement. The combination of these analyses ensured that the final version of the Ethno-STEM PjBL module was both empirically grounded and pedagogically sound.

### 2.6 Ethical considerations

Ethical considerations were addressed throughout the research process. Permission to conduct the study was obtained from the school authorities, and informed consent was secured from all participants prior to data collection. Students’ identities were kept confidential, and all data were anonymized to protect participants’ privacy. Participation in the study was voluntary, and students were free to withdraw at any stage without any academic consequences. The authors stated that the study was approved by the Institutional Review Board at Universitas Bosowa on 3 February 2025 with reference number 074c/1.02/DRIPM-UNIBOS/IV/2025. Written informed consents were obtained from the participants.

## 3. Results

### 3.1 Profile of students’ ecological awareness in the archipelago region

The exploratory phase of this study aimed to identify students’ ecological awareness profiles as a foundation for module development. Ecological awareness was measured prior to any instructional intervention using a domain-based questionnaire encompassing four dimensions relevant to archipelagic environmental contexts: consumption and carbon footprint, renewable energy, waste management, and water conservation. The results presented in this section represent students’ **baseline (pretest) ecological awareness**, reflecting their initial levels of understanding, attitudes, and self-reported behaviors related to environmental issues.

Descriptive analysis of the pretest data indicates that students’ ecological awareness across the four domains generally falls within the **moderate category**, with noticeable variation among domains. Table 2 presents the mean scores and standard deviations for each ecological awareness domain based on pretest results.

**Table 2.** Baseline profile of students’ ecological awareness.

Ecological awareness domain	Mean	SD	Category
Consumption and carbon footprint	75.00	9.94	Moderate
Renewable energy	72.00	8.91	Moderate
Waste management	71.00	7.41	Moderate
Water conservation	76.00	9.34	Moderate
**Overall ecological awareness**	**73.53**	**7.02**	**Moderate**

Overall, students demonstrated a moderate level of ecological awareness at the outset of the study. This finding suggests that students possess a basic understanding of environmental issues and show some degree of concern and environmentally responsible behavior. However, the moderate classification across all domains also indicates that ecological awareness has not yet been fully internalized or consistently translated into strong pro-environmental practices.

Among the four domains, **water conservation** showed the highest mean score, indicating relatively strong awareness related to water-saving behaviors and the importance of maintaining clean water sources. This pattern is consistent with the daily realities of island communities, where access to freshwater is limited and water use is closely monitored in household routines. Students’ familiarity with water scarcity appears to contribute to higher baseline awareness in this domain.

In contrast, **waste management** and **renewable energy** exhibited the lowest mean scores among the four domains, although both remained within the moderate category. These results suggest that while students are aware of waste-related issues and energy use at a conceptual level, their understanding and engagement may be less developed compared to water conservation practices. This condition reflects common challenges in island settings, where waste disposal systems and access to renewable energy technologies are often limited, reducing students’ direct exposure to structured environmental management practices.

The **consumption and carbon footprint** domain also showed a moderate mean score, indicating that students have some awareness of consumption patterns and their environmental impacts. However, this awareness may still be fragmented, particularly regarding abstract concepts such as carbon emissions and the broader implications of food sourcing and transportation. These findings highlight the need for learning activities that connect everyday consumption choices with ecological consequences in a more explicit and experiential manner.

### 3.2 Identification of priority ecological awareness domains for module development

To inform the design of the Ethno-STEM Project-Based Learning module, the baseline ecological awareness profile was examined to identify priority domains requiring reinforcement. Although all four domains were classified as moderate, differences in mean scores and contextual relevance provided important insights for instructional design.

The relatively higher baseline score in water conservation suggests that this domain represents an area of established awareness among students. Consequently, learning activities related to water conservation in the module were designed to reinforce and deepen existing awareness, focusing on strengthening conceptual understanding and practical skills rather than introducing entirely new behaviors.

In contrast, waste management and renewable energy emerged as priority domains for instructional emphasis. The lower baseline scores in these domains indicate gaps in students’ understanding and engagement, particularly regarding systematic waste handling, recycling practices, and the application of renewable energy solutions in daily life. These domains were therefore identified as critical entry points for project-based learning activities aimed at fostering stronger behavioral engagement and problem-solving skills.

The consumption and carbon footprint domain occupies an intermediate position, suggesting the need for learning experiences that bridge students’ everyday consumption habits with broader environmental impacts. This domain was prioritized for activities that encourage reflection, data-based analysis, and discussion of local versus non-local resource use within island contexts.

### 3.3 Implications of ecological awareness profiles for ethno-STEM PjBL module design

The ecological awareness profiles obtained from the exploratory phase directly informed the structure and content of the developed Ethno-STEM Project-Based Learning module. Each project within the module was deliberately aligned with one of the four ecological awareness domains, ensuring that learning activities addressed students’ actual needs as identified through the baseline data.

Projects related to waste management and renewable energy were designed to provide hands-on experiences and concrete problem-solving opportunities, enabling students to engage more deeply with domains that showed lower initial awareness. Meanwhile, projects focusing on water conservation emphasized contextual reinforcement and the integration of traditional knowledge to strengthen existing awareness. The consumption and carbon footprint project was structured to promote critical reflection on daily practices and their environmental implications.

By grounding module design in students’ baseline ecological awareness profiles, the Ethno-STEM PjBL module moves beyond a generic instructional approach and adopts a data-informed, context-sensitive design. This alignment ensures that learning activities are not only relevant to students’ lived experiences but also strategically targeted to support the development of ecological awareness across domains most in need of reinforcement.

### 3.4 Analysis of learning needs and project selection for ethno-STEM PjBL

To further inform the design of the Ethno-STEM Project-Based Learning module, a learning needs analysis was conducted by integrating students’ baseline ecological awareness profiles with contextual environmental issues in the archipelagic setting. This analysis aimed to identify priority domains that require instructional reinforcement and to determine project themes that are both pedagogically relevant and culturally grounded. The results of this needs analysis, along with the corresponding Ethno-STEM PjBL project selection, are summarized in Table 3.

**Table 3.** Learning needs analysis and selection of ethno-STEM PjBL projects based on students’ ecological awareness profiles.

Ecological awareness domain	Baseline awareness level (Pretest)	Identified learning needs	Selected ethno-STEM PjBL project	Rationale for project selection
Consumption and carbon footprint	Moderate	Students show general awareness of consumption issues but limited understanding of the relationship between food choices, transportation, and carbon emissions	Local Food Carbon Footprint Project	Enables students to connect daily food consumption with environmental impact using local food practices and simple carbon footprint analysis
Renewable energy	Moderate (relatively low)	Limited knowledge of renewable energy concepts and lack of exposure to practical applications	Mini Solar Power Plant Project	Provides hands-on experience with solar energy systems and links STEM concepts to local energy challenges in island contexts
Waste management	Moderate (lowest among domains)	Insufficient practical experience in waste separation, recycling, and organic waste processing	Bioconversion-Based Waste Bank Project	Encourages behavioral engagement through waste classification, composting, and community-based waste management rooted in local practices
Water conservation	Moderate (highest baseline)	Awareness already established; needs reinforcement and conceptual deepening	Traditional Water Filtration Technology Project	Reinforces existing awareness through practical application of traditional filtration methods integrated with scientific principles

Table 3 presents the results of the learning needs analysis that guided the selection of Ethno-STEM Project-Based Learning projects. The analysis integrates students’ baseline ecological awareness profiles with contextual environmental challenges commonly found in archipelagic regions. Although all four ecological awareness domains were categorized at a moderate level, variations in baseline scores and contextual relevance revealed different instructional priorities. Domains such as waste management and renewable energy demonstrated relatively lower baseline awareness and limited practical engagement, indicating a need for learning activities that emphasize hands-on experience and behavior-oriented problem solving.

The selected projects were designed to respond directly to these identified needs while maintaining strong alignment with local wisdom and STEM concepts. Projects targeting waste management and renewable energy prioritize experiential learning to strengthen domains requiring greater reinforcement, whereas the water conservation project focuses on reinforcing and deepening already established awareness through culturally grounded practices. The consumption and carbon footprint project serves as a reflective bridge between everyday habits and broader environmental impacts. This needs-based project selection ensures that the Ethno-STEM PjBL module is systematically grounded in empirical data and tailored to the ecological realities of island communities. Based on the needs analysis, a module was developed according to the predetermined theme. The module was then validated by experts, with the results shown in the Table 4.

**Table 4.** Expert validation results of learning module.

Category	Learning module		
	Average	Percentage	Category
CK (Content Knowledge)	4.69	93.75%	Valid
PK (Pedagogical Knowledge)	4.62	92.50%	Valid
TK (Technological Knowledge)	4.50	90.00%	Valid

Table 4 shows the expert validation results for the teaching modules and worksheets, which demonstrated excellent validity across the three categories tested. For both components, the teaching modules, the average scores were 4.69 for CK, 4.62 for PK, and 4.50 for TK, with validity percentages of 93.75%, 92.50%, and 90.00%, respectively. This indicates that the teaching modules are highly aligned with the learning objectives and can be relied upon to develop student knowledge, develop effective learning strategies, and utilize relevant technology.

Although the evaluation showed excellent results, the validator’s suggestions provided several directions for further improvement. The validator noted that “the distinctive local wisdom aspect needs to be deepened in the project” and that “local wisdom in the form of intangible knowledge and equipment and technology systems needs to be deepened,” demonstrating the importance of strengthening local elements in teaching materials to avoid potential misinterpretations and increase the appeal and innovation of learning. This suggestion was revised in the revised module version by adding an explicit local wisdom section to the module. Another suggestion was the need to “clarify the basis in the form of a flow or scheme for combining the elements used,” to further clarify the position of Project-Based Learning (PjBL) as a learning method. Revisions related to this were incorporated into the module/teacher learning instructions.

## 4. Discussion

The findings of this study provide important insights into how students’ ecological awareness in archipelagic contexts can be systematically addressed through data-informed instructional design. The baseline ecological awareness profile revealed that students generally demonstrated a moderate level of awareness across four domains—consumption and carbon footprint, renewable energy, waste management, and water conservation—indicating the presence of basic environmental understanding and concern, but also highlighting substantial room for further development ( Cruz & Manata, 2020). This pattern supports theoretical perspectives that conceptualize ecological awareness as a developmental construct that evolves through sustained educational experiences rather than a static attribute acquired solely through information exposure ( Hines et al., 1987).

From a theoretical standpoint, the multidimensional profile observed in this study aligns with the Hines-Hungerford-Tomera model, which emphasizes that responsible environmental behavior emerges from the interaction between environmental knowledge, affective dispositions, and situational opportunities ( Hines et al., 1987; Bamberg & Möser, 2007). Although students in this study showed moderate awareness in all domains, lower baseline scores in waste management and renewable energy suggest that cognitive awareness alone may not be sufficient to foster consistent pro-environmental behavior. This finding reinforces previous research indicating that environmental attitudes often fail to translate into action without experiential learning opportunities that make environmental issues tangible and personally relevant ( López et al., 2024).

The relatively higher baseline awareness in the water conservation domain can be interpreted through the lens of place-based learning theory ( Woodhouse & Knapp, 2000). In island communities, freshwater scarcity is a daily concern, and water-saving practices are often embedded in household routines. Such repeated exposure to environmental constraints may foster habitual awareness and behavior even in the absence of formal instruction ( Kim & Cho, 2022). Similar patterns have been reported in studies conducted in coastal and rural areas, where environmental awareness is shaped strongly by lived experience rather than classroom-based learning alone ( SMILO, 2023). However, the presence of relatively high baseline awareness does not negate the need for instructional reinforcement; instead, it suggests that learning activities should focus on deepening conceptual understanding and validating existing local practices through scientific explanation.

The learning needs analysis further demonstrates the value of using ecological awareness profiles as a foundation for instructional design. Rather than treating all environmental topics as equally urgent, this study identifies priority domains that require targeted pedagogical attention. Waste management and renewable energy emerged as domains with greater instructional needs, reflecting limited student engagement with structured waste systems and renewable technologies in daily life. These findings are consistent with prior studies showing that students tend to have lower awareness of environmental issues that are perceived as systemic or technologically complex, especially in resource-constrained settings ( Greening the Islands, 2024).

The selection of Ethno-STEM Project-Based Learning projects based on these identified needs reflects a deliberate alignment between theory, empirical data, and contextual relevance. Project-Based Learning is grounded in constructivist learning theory, which posits that learners construct knowledge most effectively when actively engaged in solving meaningful problems ( Karnoto, 2025). By integrating PjBL with STEM education and local wisdom, the Ethno-STEM approach further extends this framework by situating learning within students’ cultural and ecological contexts. Previous studies have shown that Ethno-STEM learning enhances relevance, motivation, and conceptual understanding by bridging scientific concepts with indigenous knowledge and everyday practices ( Nugraheni et al., 2023; Pranata & Setiawan, 2023).

The local food carbon footprint project exemplifies how abstract environmental concepts, such as carbon emissions, can be contextualized through culturally familiar practices. Research on environmental education consistently highlights that consumption-related behaviors are more effectively addressed when students can relate them to daily decision-making processes ( López et al., 2024). By examining local versus non-local food consumption, students are encouraged to critically reflect on the environmental implications of their choices, supporting the development of cognitive and affective components of ecological awareness.

Similarly, the mini solar power plant project responds directly to gaps identified in the renewable energy domain. Hands-on engagement with renewable energy systems aligns with experiential learning theory, which emphasizes learning through direct experience and reflection ( Pertiwi et al., 2024). Prior research on STEM-based renewable energy projects indicates that such activities not only improve conceptual understanding but also foster positive attitudes toward sustainable technologies. In archipelagic contexts where energy access is often unreliable, this project also enhances students’ perception of renewable energy as a feasible and locally relevant solution rather than a distant technological concept.

The bioconversion-based waste bank project addresses the waste management domain by emphasizing behavioral engagement and community-oriented problem solving. Waste-related behaviors are strongly influenced by social norms and practical opportunities ( Azrai et al., 2024). By involving students in waste classification, composting, and waste bank simulations, this project creates opportunities for students to practice environmentally responsible behaviors within a supportive learning environment. Previous studies have demonstrated that project-based waste management activities are particularly effective in fostering behavioral change, especially when they draw on local practices and collective responsibility ( Rohmah & Aji, 2024).

The traditional water filtration project illustrates how Ethno-STEM PjBL can reinforce existing awareness while deepening scientific understanding. Integrating traditional filtration methods with scientific explanations validates local wisdom as a legitimate source of knowledge, which is a key principle of ethnopedagogy ( Nugraheni et al., 2023; Putri & Widodo, 2025). Research on culturally responsive education suggests that acknowledging and legitimizing students’ cultural knowledge enhances engagement and strengthens identity, which in turn supports sustained learning outcomes ( Hamna & Ummah, 2024).

Overall, the findings of this study suggest that designing Ethno-STEM PjBL modules based on students’ ecological awareness profiles offers a more nuanced and responsive approach to environmental education. Rather than adopting a one-size-fits-all model, this approach allows educators to prioritize domains that require greater reinforcement while respecting existing ecological practices within the community. This strategy aligns with contemporary perspectives in Education for Sustainable Development, which emphasize contextualization, learner-centered design, and the integration of local knowledge systems ( UNESCO, 2016).

By positioning ecological awareness profiling as an integral step in instructional design, this study contributes to the growing body of research advocating data-informed and culturally grounded approaches to STEM and environmental education. The findings underscore the importance of aligning pedagogical interventions with students’ lived experiences and actual learning needs, particularly in archipelagic and resource-constrained settings where environmental challenges are both immediate and deeply embedded in daily life ( Alkhudri et al., 2024; Greening the Islands, 2024).

## 5. Conclusion

This study demonstrates that profiling students’ ecological awareness provides a strong and necessary foundation for designing context-sensitive environmental learning modules, particularly in archipelagic regions. The exploratory analysis revealed that students’ ecological awareness across the domains of consumption and carbon footprint, renewable energy, waste management, and water conservation was generally at a moderate level, with meaningful variation among domains. These findings highlight the importance of moving beyond generic environmental instruction toward learning designs that are informed by students’ actual awareness levels and lived environmental experiences.

The Ethno-STEM Project-Based Learning module developed in this study was deliberately grounded in the identified ecological awareness profiles. Each project was aligned with a specific ecological awareness domain and tailored to address domain-specific learning needs. Domains with relatively lower baseline awareness, such as waste management and renewable energy, were prioritized through hands-on and problem-oriented projects, while domains with higher baseline awareness, such as water conservation, were reinforced through contextualized and reflective activities. This domain-based design approach ensures that the module responds directly to students’ real learning needs rather than assumed deficiencies.

Despite these contributions, this study has several limitations that should be acknowledged. The ecological awareness profiling was conducted in a single archipelagic school, which limits the generalizability of the findings to other island or non-island contexts. In addition, this study focused on exploratory analysis and module development without empirically testing the effectiveness of the developed module in improving students’ ecological awareness. The reliance on self-reported questionnaire data also introduces the possibility of response bias, as students’ reported awareness may not fully reflect their actual environmental behaviors.

Future research is therefore recommended to extend this work through experimental or quasi-experimental studies that examine the effectiveness of the developed Ethno-STEM Project-Based Learning module in enhancing ecological awareness across domains. Longitudinal designs would be particularly valuable for investigating whether changes in ecological awareness are sustained over time and translated into consistent pro-environmental behaviors. Further studies could also involve multiple schools or different archipelagic regions to explore how contextual factors, such as cultural practices and resource availability, influence the effectiveness of Ethno-STEM–based environmental learning. By building on the diagnostic and design-oriented approach proposed in this study, future research can contribute to more robust and scalable models of ecological education in diverse educational settings.

## Ethical consideration

The authors stated that the study was approved by the Institutional Review Board at Universitas Bosowa on 3 February 2025 with the reference number 074c/1.02/DRIPM-UNIBOS/IV/2025. Written informed consents were obtained from the participants.

## AI statement

The authors stated that generative artificial intelligence tools were used in a limited manner to assist with language editing, organization of academic writing, and improvement of clarity. The conceptual framework, research design, data analysis, interpretation, and final conclusions were developed entirely by the authors.

## Data Availability

The data supporting the findings of this study are available in figshare:
• Questionnaire of Ecological Awareness. DOI:
https://doi.org/10.6084/m9.figshare.31396434 ( Hamid, 2026a)
• Module Expert Validation Sheet. DOI:
https://doi.org/10.6084/m9.figshare.31396524 ( Hamid, 2026b). Data are available under the terms of the Creative Commons Attribution 4.0 International license.

## References

[ref1] Alkhudri AT Heryanto N Gustiannisa S : Ecopedagogy of Island Communities: Conception, Planning to Action in Building Environmental Awareness in Kelapa Island and Harapan Island, Administrative District Kepulauan Seribu-DKI Jakarta. *KnE Social Sciences.* 2024;472–486.

[ref2] Azrai EP Heryanti E Ramadhani V : Enhancing students’ pro-environmental behavior through project-based learning. *Biosfer: Jurnal Pendidikan Biologi.* 2024;17(2):317–325. 10.21009/biosfer.17.2.8

[ref3] Bamberg S Möser G : Twenty years after Hines, Hungerford, and Tomera: A new meta-analysis of psycho-social determinants of pro-environmental behaviour. *J. Environ. Psychol.* 2007;27(1):14–25. 10.1016/j.jenvp.2006.12.002

[ref4] Babintseva E Mansur D Trifonova E : Impact of Environmental Education Development on Promoting Environmentally Safe Use of Natural Resources And Careful Attitude Toward Nature. *International journal of ecosystems and ecology science (IJEES).* 2025;15(1):123–132. 10.31407/ijees15.114

[ref5] Bock HW Wickings KG : Cultivating ecological awareness in middle-school students through short interactions with urban soil ecosystems. *Urban Ecosyst.* 2025;28(3):120. 10.1007/s11252-025-01736-0

[ref6] Cruz SM Manata B : Measurement of environmental concern: A review and analysis. *Front. Psychol.* 2020;11. 10.3389/fpsyg.2020.00363 32210883 PMC7067970

[ref7] Dipalaya T Pratiwi AC Muriati S : Ecological Awareness and Behaviour among University Students in Education Majors: an Empirical Study. *IOP Conference Series: Earth and Environmental Science.* IOP Publishing;2024, December; Vol.1425(1): p.012016.

[ref8] Greening the Islands: GTI Observatory Report – Water: Biggest threat and opportunity for islands' sustainability. 2024.

[ref9] Guo P Saab N Post LS : A review of project-based learning in higher education: Student outcomes and measures. *Int. J. Educ. Res.* 2020;102:101586. 10.1016/j.ijer.2020.101586

[ref10] Hamna H Ummah MK : The effectiveness of ethnoscience learning based on local wisdom values in elementary schools. *Madako Elementary School Journal.* 2024;3(2):165–183.

[ref11] Hamid S : Questionnaire of Ecological Awareness.Dataset. *figshare.* 2026a. 10.6084/m9.figshare.31396434.v2

[ref12] Hamid S : Module Expert Validation Sheet (English Version).Dataset. *figshare.* 2026b. 10.6084/m9.figshare.31396524.v2

[ref13] Hines JM Hungerford HR Tomera AN : Analysis and synthesis of research on responsible environmental behavior: A meta-analysis. *J. Environ. Educ.* 1987;18(2):1–8. 10.1080/00958964.1987.9943482

[ref14] Karnoto BK : The impact of project-based learning on students' science literacy and environmental awareness: A comprehensive review. *J. Pedagogi.* 2025;6(1):1–18. Reference Source

[ref15] Kim M Cho H : Exploring place-based environmental education for participatory citizens. *The Journal of the Korean Association of Geographic and Environmental Education.* 2022;30(3):33–53.

[ref16] López JA Vizcaíno-Verdú A García-Escudero MP : Effects of a project-based learning methodology on environmental awareness of secondary school students. *Int. J. Instr.* 2024;17(1):1–22. 10.29333/iji.2024.1711a

[ref17] Martawijaya MA Rahmadhanningsih S Swandi A : The effect of applying the Ethno-STEM-Project-based learning model on students' higher-order thinking skill and misconception of physics topics related to Lake Tempe, Indonesia. *Jurnal Pendidikan IPA Indones.* 2023a;12(1):1–13. 10.15294/jpii.v12i1.38703

[ref18] Martawijaya MA Swandi A Rahmadhanningsih S : Development of student worksheets with the ethno-STEM-project based learning model on physics concepts related to Danau Tempe. *The 8th International Conference On Mathematics, Science And Education 2021.* AIP Publishing LLC;2023b, June; Vol.2614(1): p.050075.

[ref19] Masriah I Rachman I Ardianto D : Assessing the validity and reliability of environmental awareness measurement tools in agricultural area school. *Int J Sci Adv.* 2025;6(3):550–562. 10.51542/ijscia.v6i3.55

[ref20] Nugraheni FSA Sari MW Wati IK : Indigenous knowledge and its potential for junior high school ethno-STEM learning. *AIP Conference Proceedings.* 2023;2540. Article 110010. 10.1063/5.0109475

[ref21] Pertiwi TU : The effectiveness of STEM project-based learning in improving students' environmental literacy abilities. *Jurnal Pendidikan Biol Indones.* 2024;10(2):192–204. 10.22219/jpbi.v10i2.33562

[ref22] Pranata A Setiawan D : Development of ethno-STEM e-module with project-based learning model based on local wisdom of Yogyakarta to increase students' creativity. *Int J Res Rev.* 2023;10(10):92–102.

[ref23] Putri DK Widodo W : Case study: Ethno-STEM based learning to enhance critical thinking skills in primary school students. *J Innov Res Prim Educ.* 2025;4(3):724–732. 10.56916/jirpe.v4i3.1442

[ref24] Rohmah S Aji M : Ethno-STEM integrated project-based learning to improve students' creative thinking skills. *Int J Ethnosci Technol Educ.* 2024;1(2):116–130. 10.33394/ijete.v1i2.11308

[ref25] SMILO (Small Islands Organisation): Water and sanitation on small islands. 2023. Reference Source

[ref26] Sudarmin S Pujiastuti RSE Asyhar R : Chemistry project-based learning for secondary metabolite course with ethno-STEM approach to improve students' conservation and entrepreneurial character in the 21st century. *JOTSE.* 2023;13(1):393–409. 10.3926/jotse.1792

[ref27] Sumarni W Kadarwati S : Ethno-stem project-based learning: Its impact to critical and creative thinking skills. *Jurnal Pendidikan IPA Indonesia.* 2020;9(1):11–21. 10.15294/jpii.v9i1.21754

[ref28] UNESCO: *Education for sustainable development goals: Learning objectives.* UNESCO;2016. Reference Source

[ref29] UNESCO: *A decade of progress on education for sustainable development: Reflections from the UNESCO Chairs Programme.* UNESCO;2017.

[ref30] UNESCO: *Improving knowledge of water security and sustainable living for local communities.* UNESCO Office Jakarta and BRIN;2023. Reference Source

[ref31] UNESCO: *Education for sustainable development: Learning to act for people and planet.* UNESCO;2025. Reference Source

[ref32] Woodhouse JL Knapp CE : *Place-based curriculum and instruction: Outdoor and environmental education approaches (ERIC Digest No. ED448012).* ERIC;2000. Reference Source

[ref33] Yusoff MSB : ABC of content validation and content validity index calculation. *Educ Med J.* 2019;11(2):49–54. 10.21315/eimj2019.11.2.6

[ref34] Zamanzadeh V Ghahramanian A Rassouli M : Design and implementation content validity study: Development of an instrument for measuring patient-centered communication. *J. Caring Sci.* 2015;4(2):165–178. 10.15171/jcs.2015.017 26161370 PMC4484991

