# Supplementary material for: Exploration of Local Wisdom of the Archipelago Region to Design Ethno-STEM Project-Based Learning Modules for Ecological Awareness Learning
Source: F1000Res. 2026 Mar 18;15:411. [Version 1] doi: 10.12688/f1000research.178677.1 (PMC13062768; doi:10.12688/f1000research.178677.1)
Supplement: Peer review history

# Reviewer report: Reviewer response for version 1

**DOI:** 10.5256/f1000research.197093.r469407

**Published:** 2026-04-08

**Authors:** Ida Sriyanti

**Affiliations:** 1 Physics Education, Universitas Sriwijaya, Palembang, South Sumatra, Indonesia

This article explores the creation of an Ethno-STEM module informed by the ecological awareness profile of students in an Indonesian archipelago. The work is generally well-presented and backed by current literature. While the research design is pertinent, it requires further refinement, particularly regarding methodological consistency and the depth of exploration into local wisdom. Clarification is needed for instrument descriptions, interview procedures, and validity analysis to ensure replicability. The conclusions should be strictly based on the data to avoid overstatements. The author recommends incorporating more methodological details, enhancing the empirical integration of local wisdom, and ensuring alignment between the abstract, methods, and results to enhance the article's scientific rigor.

Is the work clearly and accurately presented and does it cite the current literature?

Yes

If applicable, is the statistical analysis and its interpretation appropriate?

Yes

Are all the source data underlying the results available to ensure full reproducibility?

Yes

Is the study design appropriate and is the work technically sound?

Yes

Are the conclusions drawn adequately supported by the results?

Yes

Are sufficient details of methods and analysis provided to allow replication by others?

Yes

Reviewer Expertise:

Physics Education

I confirm that I have read this submission and believe that I have an appropriate level of expertise to confirm that it is of an acceptable scientific standard.

# Reviewer report: Reviewer response for version 1

**DOI:** 10.5256/f1000research.197093.r469406

**Published:** 2026-04-07

**Authors:** Syahmani Syahmani, Arif Sholahuddin

**Affiliations:** 1 Universitas Lambung Mangkurat, Banjarmasin, South Kalimantan, Indonesia; 2 Science Education, Universitas Lambung Mangkurat, Banjarmasin, South Kalimantan, Indonesia

Abstract - This study aimed to profile students’ ecological awareness and use the findings as a basis for designing an Ethno-STEM Project-Based Learning (Ethno-STEM PjBL) module.

 Methods “An exploratory and design-oriented approach” sounds good, but methodologically, it remains too general. If there is a profiling stage followed by module development, it can be called exploratory sequential design.

 Keywords make it simple: Ethno-STEM; project-based learning; ecological awareness; local wisdom; archipelagic education

 Introduction: The research gap. It's unclear whether the problem is low ecological awareness, disparities between domains, or the lack of locally context-based modules.

 Science learning in island regions often lacks context-sensitive materials and rarely integrates local ecological knowledge effectively.

 Method: Presentation and reasoning of research methodology and methods appropriate

 The research results highlight ecological awareness at a moderate level, showing significant variation across domains, but arguments regarding specific strengths and weaknesses between these domains are less clear.

 Discuss: Depth of scientific discussion, level of argumentation, and value appropriate.

 The conclusion already shows theoretical and practical contributions.

 Overall quality of the article is good.

Is the work clearly and accurately presented and does it cite the current literature?

Yes

If applicable, is the statistical analysis and its interpretation appropriate?

Yes

Are all the source data underlying the results available to ensure full reproducibility?

Yes

Is the study design appropriate and is the work technically sound?

Partly

Are the conclusions drawn adequately supported by the results?

Yes

Are sufficient details of methods and analysis provided to allow replication by others?

Yes

Reviewer Expertise:

Expertise/specializing in STEM/STEAM, HOTSEP, metacognition, misconceptions, and pedagogical innovations.

We confirm that we have read this submission and believe that we have an appropriate level of expertise to confirm that it is of an acceptable scientific standard.

# Reviewer report: Reviewer response for version 1

**DOI:** 10.5256/f1000research.197093.r469404

**Published:** 2026-04-07

**Authors:** Hartono Bancong

**Affiliations:** 1 Universitas Muhammadiyah Makassar, Makassar, South Sulawesi, Indonesia

The manuscript is generally clear and logically structured, and it cites a number of recent references alongside foundational literature. The study design is acceptable for an exploratory-developmental study, and the results are presented in a way that shows how baseline ecological-awareness data informed the module design. However, several issues should be addressed.

 1. The title foregrounds the “exploration of local wisdom,” yet the reported empirical core of the paper is students’ ecological-awareness profiling, not a systematic exploration of local wisdom. The manuscript does mention the concept of local wisdom conceptually and embeds it in the project’s rationale, but the methods and results do not present a clear, stand-alone process for identifying, documenting, and analyzing local wisdom in the archipelagic community. This becomes especially important because the validators themselves noted that the local-wisdom aspect needed to be deepened. To make the manuscript scientifically sound, the authors should either: (a) add a clear empirical local-wisdom component, for example, through interviews with community members, teachers, or local practitioners, observations, or cultural artifact analysis; or (b) revise the title and claims so they accurately reflect that the study primarily profiles ecological awareness and proposes locally informed module designs, rather than fully “exploring” local wisdom.

 2. There is a methodological inconsistency regarding interviews. The abstract states that data were collected using validated questionnaires and interviews. However, the Methods section primarily describes the questionnaire and expert validation sheet and does not provide adequate details about interviews: who was interviewed, how many participants were interviewed, how interview guides were developed, how the data were analyzed, and how interview findings contributed to the module design. This inconsistency must be resolved. If interviews were conducted, they must be fully described and reported. If they were not central to the study or not analyzed systematically, they should be removed from the abstract and other relevant sections.

 3. The ecological-awareness instrument is underreported. The paper states that the questionnaire covers four domains and includes cognitive, affective, and behavioral aspects, but it does not provide enough detail for readers to evaluate or replicate the measurement. The manuscript should report the number of items per domain, example items, scoring scale, score range, whether any items were reverse-coded, how domain and overall scores were computed, and how the “moderate” category thresholds were determined. The statement that the instrument was “validated” also requires explicit psychometric evidence, such as content validity procedures and reliability estimates for the sample or for prior validated versions. Without this information, the reported means and the interpretation of “moderate” awareness remain difficult to assess.

 4. The validation procedure and reporting need substantial clarification. The methods section states that I-CVI and S-CVI were calculated, but the results table presents averages and percentages for CK, PK, and TK. It is not clear how these categories map onto the stated CVI approach, how many experts were involved, what rating scale was used, or how the percentages were calculated. The abstract also states that content validity indices were “>0.90 across components,” yet one reported percentage is 90.00%, which equals 0.90 rather than being greater than 0.90. This should be corrected for precision. To make this section sound, the authors should specify the number and expertise of validators, report the exact formulas and decision criteria for I-CVI/S-CVI, define CK/PK/TK explicitly, and present the item-level and/or scale-level validity results consistently with the stated method.

 5. The conclusions should be narrowed. The paper is justified in concluding that ecological-awareness profiling can inform module design and that the resulting module achieved good expert-feasibility judgments. However, the conclusions should not imply demonstrated impact on ecological awareness learning, because there was no implementation, no pre-post evaluation of the module, and no comparison group. The manuscript itself acknowledges this limitation, and the conclusion should align more strictly with it.

Is the work clearly and accurately presented and does it cite the current literature?

Partly

If applicable, is the statistical analysis and its interpretation appropriate?

Partly

Are all the source data underlying the results available to ensure full reproducibility?

Partly

Is the study design appropriate and is the work technically sound?

Partly

Are the conclusions drawn adequately supported by the results?

Partly

Are sufficient details of methods and analysis provided to allow replication by others?

Partly

Reviewer Expertise:

Science education; STEAM education, instructional design

I confirm that I have read this submission and believe that I have an appropriate level of expertise to confirm that it is of an acceptable scientific standard, however I have significant reservations, as outlined above.
